# Neuronal characteristics of small-cell lung cancer

**DOI:** 10.1038/sj.bjc.6602857

**Published:** 2005-11-01

**Authors:** P U Onganer, M J Seckl, M B A Djamgoz

**Affiliations:** 1 Division of Cell and Molecular Biology, Imperial College London, South Kensington Campus, London SW7 2AZ, UK; 2 Cancer Medicine, Imperial College London, Hammersmith Campus, London SW7 2AZ, UK

**Keywords:** small-cell lung cancer, neuronal markers, voltage-gated ion channels

## Abstract

Wide ranging experimental evidence suggests that human small-cell lung cancer (SCLC) has a number of molecular and subcellular characteristics normally associated with neurones. This review outlines and discusses these characteristics in the light of recent developments in the field. Emphasis is placed upon neuronal cell adhesion molecules, neurone-restrictive silencer factor, neurotransmitters/peptides and voltage-gated ion, especially Na^+^ channels. The hypothesis is put forward that acquisition of such characteristics and the membrane ‘excitability’ that would follow can accelerate metastatic progression. The clinical potential of the neuronal characteristics of SCLC, in particular ion channel expression/activity, is discussed in relation to possible novel diagnostic and therapeutic modalities.

Small-cell lung cancer (SCLC) has the most aggressive clinical course of any type of pulmonary tumour, with median survival time from diagnosis of only months. This is a ‘neuroendocrine’ tumour for which localised forms of treatment, such as surgical resection or radiation therapy, rarely produce long-term cure, and cytotoxic chemotherapy remains the main method of treatment. A remarkable feature of SCLC is that it is the most commonly encountered form of neoplasm associated with a wide range of paraneoplastic neurological syndromes (PNSs), including Lambert–Eaton myasthenic syndrome (LEMS), and in more rare cases, cerebellar degeneration, encephalomyelitis and sensory neuropathy (Honnorat and Cartalat-Carel, 2004). These are a heterogeneous group of disorders that are due to secretion of and/or autoimmune responses to specific molecules produced by the cancer cells, rather than any direct invasion by tumours. Lambert–Eaton myasthenic syndrome, which is a form of muscle weakness, is well understood and is due to the SCLC tumour expressing voltage-gated Na^+^ and/or Ca^2+^ channels. In response, the immune system produces autoantibodies against the channel proteins, which in turn, can result in suppression of activity at neuromuscular junctions (Pancrazio *et al*, 1989). Such effects can also cause degeneration of central nervous system (CNS) structures, particularly Purkinje cells, which have very elaborate dendritic trees active in Ca^2+^ signalling. Voltage-gated Na^+^ and/or Ca^2+^ channels (VGSCs and VGCCs) are commonly associated with ‘excitable’ cells but also occur in a variety of non-neuronal cell types (e.g. Diss *et al*, 2004).

A recent functional ‘neuroscience’ approach that we designed to elucidate the pathophysiology of human cancer revealed that membranes of metastatic prostate and breast cancer cells are potentially ‘excitable’, involving concomitant expression of high levels of VGSC and reduced levels of voltage-gated K^+^ channel (e.g. Laniado *et al*, 1997; Fraser *et al*, 2005). The purpose of this review is to outline and evaluate the various neuronal aspects of SCLC, in an integrated approach, with a view ultimately to questioning their possible functional consequences and clinical potential.

## ‘CLASSIC’ MARKERS

A number of antineuronal nuclear antibodies and neurogenetic markers are well established to be associated with human SCLC. The term ‘antineuronal nuclear antibody’ (ANNA) was used originally in relation to the sensory neuropathy syndrome associated with SCLC. Three main ANNAs have been found, ANNA-3 being most closely related to SCLC. The ‘neurogenetic markers’ compromise the four Hu antigen proteins, HuR, HuD, HuC and Hel-N1 of which HuC, HuD and Hel-N1 are expressed in neuronal tissues and SCLCs. The Hu antigens have a crucial role in the development and maintenance of the neuronal phenotype, but their function(s) in SCLC is unknown. It is possible that anti-Hu antibodies are part of a complex immune response against Hu antigens that initially target tumour growth but are misdirected to cause neurological dysfunction.

Enolases are a group of glycolytic enzymes that exist as dimers and have broad involvement in mammalian tissue metabolism. Of the two main isoforms found, one is termed ‘neurone specific enolase’ (NSE). High concentrations of NSE have been detected in neuroendocrine cells and neurogenic tumours, as well as in the blood of SCLC patients. Indeed, NSE is produced by lung carcinomas and is considered a characteristic tumour-marker in SCLC. Untreated patients with extensive disease (both lungs affected or disease detected outside lungs) had higher serum NSE levels than patients with only localised tumour.

Aromatic L-amino acid decarboxylase (AADC) is an enzyme of the lyase class that catalyses the decarboxylation of aromatic amino acids, converting dopa to dopamine, tryptophan to tryptamine, and hydroxytryptophan to serotonin. This enzyme is particularly abundant in brain and other organs such as liver, kidney and vas deferens. The activity of AADC has been measured in various normal and tumour cells and tissues and a high level of mRNA expression and activity were found in SCLC.

Chromogranin A (CgA) has been shown to regulate secretory granule formation and is coreleased with amines and neuropeptides. Detection of CgA in tumour tissue is associated with poor prognosis. The ubiquitous presence of CgA in ‘neuroendocrine’ cells/tissues makes it a suitable circulating marker for SCLC. It is not known whether NESP55, a novel Cg-like acidic secretory protein, that has also been associated with endocrine tumours, also occurs in SCLC.

## NEURONAL CELL ADHESION MOLECULES

Tumour cells generally express numerous cell adhesion molecules (CAMs), since adhesion, detachment and aggregation play an important role in tumour invasion and metastasis. Of the two groups of CAMs that have particularly been well-characterised (cadherins and Ig superfamily), the neuronal CAM (NCAM), belonging to the latter, has also been found to be expressed on the surface of SCLC cells. Neuronal CAMs are encoded by one gene but three major isoforms are produced by mRNA splicing, giving rise to proteins of 120, 140 and 180 kDa molecular weight. Each NCAM is extensively modified by post-translational glycosylation; in the developing embryo, NCAMs have relatively high sialic acid content (30% by molecular weight *vs* 10% in the adult) and this results in lower affinity between NCAMs in their homophilic binding. Neuronal CAM appears early in embryonic cells and is important in the formation of cellular assemblies and their boundaries at sites of morphogenesis. Later in development, NCAM is found on various differentiated tissues, mediating adhesion among neurones and between nerve and muscle. Expression of NCAM is highly indicative of neuroendocrine differentiation and is a potential tumour marker for SCLC (e.g. Ledermann *et al*, 1994).

## NEURONE-RESTRICTIVE SILENCER FACTOR

The ‘neurone-restrictive silencer factor’ (NRSF), also called ‘REST’, is a ‘master regulator’, repressing transcription of key neuronal genes in non-neuronal cells through the ‘neurone-restrictive silencer element’ (NRSE). The relevant motif occurs in many ‘neuronal’ genes and NRSE has been identified close to the transcriptional start site of the vasopressin promoter. In SCLC, this binds multiple NRSF-related complexes that may antagonise the normal repressor function and thus contribute to the neuroendocrine differentiation of SCLC cells. A high level of expression of a splice variant of NRSF (sNRSF) has been identified in SCLC (Ledermann *et al*, 1994). The biological role of NRSF and its specific expression in SCLC remain to be characterised, but two main possibilities may be considered. First, NRSF-mediated transcriptional silencing may be involved in specific expression of neuropeptides acting as autocrine growth factors in SCLC (Quinn *et al*, 2002). Second, NRSF/REST may regulate ion channel expression. In particular, the VGSC expression/activity, associated closely with SCLC (Pancrazio *et al*, 1989; Blandino *et al*, 1995; Onganer and Djamgoz, 2005), has been shown to be suppressed by NRSF/REST (Chong *et al*, 1995). Importantly, a recent study has identified REST as a tumour suppressor and has shown that a defective REST gene can be tumourigenic (Westbrook *et al*, 2005).

## VOLTAGE-GATED ION CHANNELS

**Figure 1:**
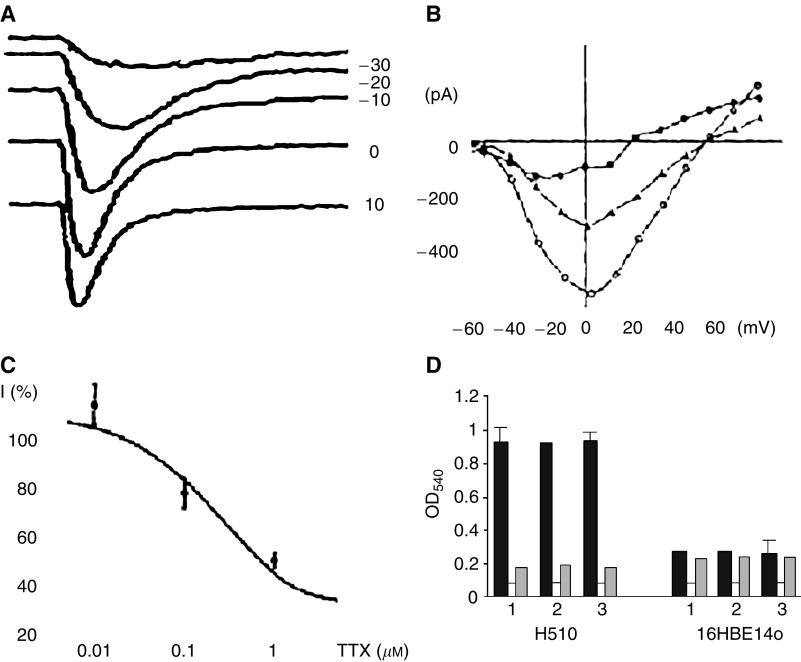
Whole-cell patch-clamp recordings from SCLC H146 cells showing expression voltage activated inward (Na^+^) currents. (**A**) A family of membrane currents activated at different membrane potentials (values in mV indicated on the right). (**B**) Current–voltage relationship of voltage-gated Na^+^ currents. Circles – normal data; triangles – effect of 100 nM TTX; squares – effect of Na^+^-free medium (choline^+^ used as substitute). (**C**) Dose–response curve for TTX-induced suppression of the Na^+^ current, *I* (%) expressed as a percentage of the control value. (**A–C**) Modified from Blandino *et al* (1995). (**D**) Effects of 100 nM TTX (1), 200 nM lidocaine (2) and 200 nM phenytoin (3) on endocytic membrane activity (HRP uptake) into the SCLC cell line, H510 (left-hand sets of histobars) and the normal airway epithelial cell line, 16HBE14o (right-hand sets of histobars). OD_540_, optical density of HRP content of cell lysates. Each data set of three histobars shows the effects of the following: HRP uptake (dark), endogenous peroxidase activity (white) and drug (grey) – TTX (1), lidocaine (2) or phenytoin (3). Each histobar represents the average±s.d. of data from at least six experiments. (**D**) Modified from Onganer and Djamgoz (2005).

Voltage-activated ion channels are a hallmark of neuronal excitability. In particular, VGSCs are necessary for initiation and conduction of regenerative potentials and VGCCs are frequently involved in secretion. Ion channel activity can be controlled by mitogens and oncogenes, and itself can affect metastatic cell behaviour, including proliferation. A high level VGSC and VGCC expression and electrophysiological activity, similar to those in excitable tissues, have been associated with human SCLC cells (Pancrazio *et al*, 1989; Blandino *et al*, 1995) (Figure 1A and B). These VGSCs appeared to be a mixture of tetrodotoxin (TTX)-sensitive and TTX-resistant channels, with a net IC_50_ of ∼100 nM (Blandino *et al*, 1995; Figure 1B and C). However, the functional role that these channels could play in SCLC behaviour is unknown. Interestingly, similar upregulation of VGSC expression/activity has been found in human metastatic prostate cancer *in vitro* (Laniado *et al*, 1997) and *in vivo* (Diss *et al*, 2005). Moreover, blockage of VGSC activity by the highly specific TTX suppressed a variety of cellular behaviours that would be involved in the metastatic cascade, including process extension, directional motility (e.g. Djamgoz *et al*, 2001), secretory membrane activity (e.g. Krasowska *et al*, 2004), adhesion (Mycielska *et al*, 2004), gene expression (e.g. Mycielska *et al*, 2005) and invasiveness *in vitro* (e.g. Laniado *et al*, 1997). Emerging data suggest that there is a comparable situation in metastatic human breast cancer cells (Fraser *et al*, 2005).

**Figure 2:**
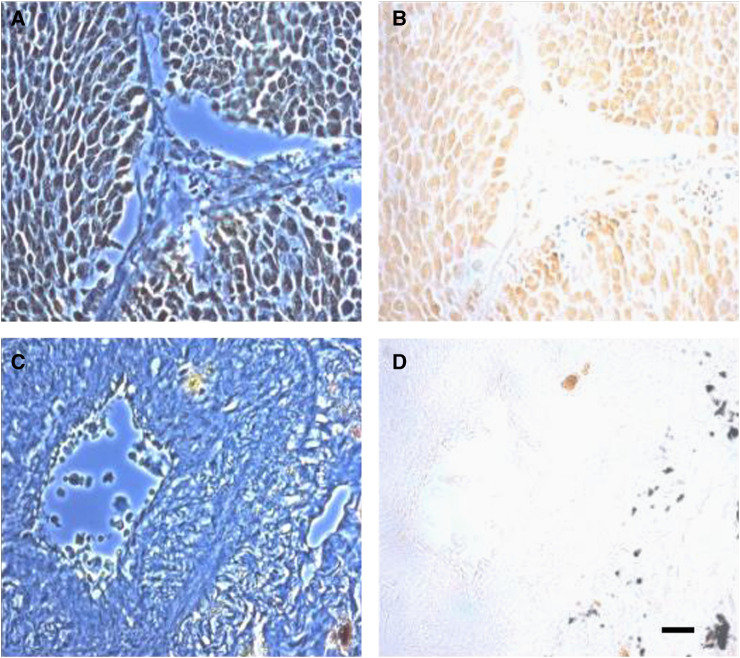
Immunohistochemical localisation of voltage-gated Na^+^ channel (VGSC) protein expression in human lung tissues, using a commercial pan-VGSC antibody. (**A** and **B**) SCLC, (**C** and **D**), normal lung epithelia. Voltage-gated Na^+^ channel immunoreactivity showed marked upregulation in SCLC (**B** *vs* **D**). Left hand panels (**A** and **C**) represent phase contrast images, right hand panels (**B** and **D**) are bright field images. Scale bar, 15 *μ*m, applicable to all parts of the figure.

The possible involvement of VGSC activity in metastatic behaviour of human SCLC cells has recently been investigated by determining their role in endocytic membrane activity, a measure of vesicular secretion and plasma membrane protein turnover (Onganer and Djamgoz, 2005) and proliferation (Onganer *et al*, 2004). A variety of human SCLC cell lines (H69, H209 and H510) and a normal human airway epithelial (16HBE14o) cell line were used. Endocytic uptake of a noncytotoxic tracer, horseradish peroxidase (HRP) into SCLC cells was vesicular and was inhibited significantly by TTX, as well as clinically used VGSC drugs, lidocaine and phenytoin (Figure 1D). These effects were dose dependent. None of the VGSC blockers used had any effect on tracer uptake into the 16HBE14o cells (Onganer and Djamgoz, 2005). Treatment with TTX for 24 h caused ∼60% significant reduction in proliferation of SCLC but only ∼15% in normal airway epithelial cells (Onganer *et al*, 2004). These data would suggest strongly that VGSC upregulation could enhance metastatic cell behaviour in SCLC, as shown previously for prostate and breast cancer. Voltage-gated Na^+^ channel protein expression in human clinical biopsies of SCLC was studied by immunohistochemical staining using a pan-VGSC antibody. There was little or no VGSC protein expressed in normal human lung tissues (Figure 2C and D). On the other hand, significant upregulation of VGSC protein was seen in SCLC (Figure 2A and B) (Onganer *et al*, 2004).

## NEUROTRANSMITTERS/PEPTIDES AND RECEPTORS

An increasing number of neuropeptides, including bombesin or gastrin releasing peptide, bradykinin, vasopressin, galanin, neurotensin, gastrin and cholecystokinin have been implicated in driving the proliferation of certain SCLC cell lines in an autocrine/paracrine fashion (reviewed by Seckl and Rozengurt, 1998). These short regulatory peptides bind to specific cell surface receptors belonging to the seven-transmembrane domain receptor superfamily. Ligand-bound receptors stimulate heterotrimetric G-proteins on their intercellular surfaces to elicit downstream signalling controlling proliferation. Neuropeptides are also known to modulate VGSCs (e.g. Montano and Djamgoz, 2004). As noted above, the latter may directly regulate growth or alternatively can trigger the further secretion of growth factors from SCLC cells. Stimulation of SCLC growth by acetylcholine or muscarine (exogenous or endogenous) could be due to Ca^2+^ influx mediated by ‘neuronal’ (*α*7) subtype of nicotinic cholinergic receptor activation and/or the subsequent opening of VGCCs, triggering autocrine release of growth factors and/or transcription of growth-regulatory genes (Song *et al*, 2003).

Glutamate is the major excitatory neurotransmitter in the mammalian CNS. A transcriptional gene expression (microarray) profiling study revealed occurrence of ‘fast-acting’ ionotropic glutamate receptors (iGluRs) in SCLC cells (Pedersen *et al*, 2003). At present, the role of iGluR expression in SCLC is not known. During neuronal development, glutamate receptors control proliferation and migration. Glutamate receptor antagonists were found to inhibit proliferation and motility and increase cell death in lung carcinomas and a variety of other cancers, and glutamate antagonists enhanced the effects of cytotoxic drugs (Rzeski *et al*, 2002).

## CONCLUSIONS AND FUTURE PERSPECTIVES

Liotta and Clair (2000) have commented that ‘… cancer invasion in general may be a deregulated form of a physiological invasion process required for neuronal wiring in the embryo, tissue remodelling of blood vessels, and healing’. The overall conclusion of the present review is that SCLC has a variety of neuronal characteristics and that SCLC cells are ‘excitable’. These characteristics have been studied individually over the years, but clusters of neuronal/neuroendocrine genes have also been detected by more recent microarray analyses (Sugita *et al*, 2002; Pedersen *et al*, 2003). Currently, there are two important questions to consider:

First, why should SCLC and other carcinoma, derived from epithelial cells, acquire such ‘neuronal’ characteristics? In the case of SCLC, in the first instance, such a strong parallel may not appear so surprising considering that SCLC cells are derived from neuroectoderm. More intriguingly, however, this could be due to neuronal mechanisms being appropriate for membrane ‘excitability’ and hyperactive cell behaviour, hallmarks of metastasis. As already noted, upregulation of voltage-gated ion channel activity potentiates a range of cell behaviours integral to the metastatic cascade (e.g. Mycielska *et al*, 2004; Fraser *et al*, 2005; Onganer and Djamgoz, 2005). There is no doubt that this would be aided critically by the force of the cells’ membrane potential (equivalent to some 10^7^ V m^−1^) and rate of ionic permeation through the voltage-gated ion channels (∼10^5^ ions ms^−1^, in single-file). Interestingly, in SCLC, prognosis was found to be better with LEMS than without, implying that the VGSC/VGCC autoantibodies may have some protective effect, consistent with the proposed role of channel activity in the metastatic cascade (Maddison *et al*, 1999).

Second, why does the immune system recognise the expression of at least some of these neuronal antigens as ‘foreign’ and give rise to PNSs? One possible answer is that such genes are expressed differently to their normal adult form, possibly due to the de-differentiated nature of cancer tissue. Importantly, in some instances, the underlying genes have been found to be ‘embryonic’ splice variants, consistent with the notion of the (re)expression of oncofeotal genes in cancer (e.g. Monk and Holden, 2001). In fact, VGSC and NCAM genes expressed in carcinomas were found to be ‘oncofeotal’ (Pedersen *et al*, 2003; Diss *et al*, 2005; Fraser *et al*, 2005).

It is likely that SCLC cells share additional properties with neurons. A particularly interesting mechanism is Hedgehog signalling, which immediately precedes the neuroendocrine differentiation in SCLC (Watkins *et al*, 2003). Hedgehog signalling also plays a significant role in the patterned growth of CNS. Another characteristic is expression of neuronal transcription factors (e.g. POU) in SCLC (Leblond-Francillard *et al*, 1997).

Generally, SCLC is so aggressive that metastasis would have occurred by the time it is detected and current therapy methods (chemotherapy, radiotherapy and surgery) are of limited effect. Consequently, there is an urgent need to develop new methods for its clinical management. A final question, therefore, is whether the neuronal characteristics of SCLC are of diagnostic and/or therapeutic value. Diagnoses based upon some neuronal characteristics (e.g. NCAM) so far have given mixed results (Chua *et al*, 2004). As regards possible therapy, a previous attempt targeting NCAM was also disappointing (Murray *et al*, 2004). An interesting future possibility would be to test the diagnostic and therapeutic potential of functional voltage-gated ion channels (VGSC and/or VGCC) expressed in SCLC. If these were a common focus of signalling downstream of multiple growth factors, VGSC/VGCC expression could be a particularly attractive target, since inhibitors of individual receptor mechanisms are unlikely to be successful given the large array of distinct growth factors and receptors involved in SCLC growth. The indications from cancers of prostate and breast, as well the data emerging from SCLC (Onganer *et al*, 2004; Onganer and Djamgoz, 2005) are that the nature of the VGSC expression/involvement in metastatic cell behaviour is so as to make it an early, functional marker of metastatic disease. Furthermore, since TTX or anticonvulsant drugs inhibit metastatic cell behaviour, VGSCs could represent a novel target for suppressing SCLC.
